# Effect of partial exchange of lactose with fat in milk replacer on performance and blood metabolites of Holstein calves

**DOI:** 10.3168/jdsc.2022-0231

**Published:** 2022-10-28

**Authors:** Juanita Echeverry-Munera, Liliana Amado, Harma Berends, Leonel N. Leal, Michael A. Steele, Javier Martín-Tereso

**Affiliations:** 1 Department of Animal Biosciences, University of Guelph, Guelph, ON, Canada N1G 2W1; 2 Trouw Nutrition R&D, PO Box 299, 3800 AG, Amersfoort, the Netherlands; 3 Animal Nutrition Group, Wageningen University, PO Box 338, 6700 AH, Wageningen, the Netherlands

## Abstract

The objective of this study was to determine the effect of dietary energy source (fat vs. carbohydrate) in calf milk replacer (MR) on growth performance parameters and feed intake in rearing calves. In a randomized complete block design, 68 Holstein calves [40 females and 28 males; (mean ± SD) body weight (BW): 43.7 ± 1.43 kg] were assigned to 17 blocks of 4 calves based on birth date and parity of the dam. Within each block, calves were randomly assigned to 1 of 2 treatments: a high-lactose MR (HL; 17% fat; 44% lactose; n = 34), or a high-fat MR (HF; 23% fat; 37% lactose; n = 34). Lactose was exchanged for fat on a weight per weight basis, resulting in a 6% difference in metabolizable energy density per kilogram of MR. The feeding plan started with 6 L/d for 7 d, then 8 L/d for 35 d, 6 L/d for 7 d, and finally, 4 L/d for 7 d. Milk replacer allowances were offered in 2 meals per day at 140 g/L. Measurements included daily MR, starter and straw intakes, weekly BW, and blood metabolites, including nonesterified fatty acids (NEFA) and glucose, on wk 4, 6, 8, and 10. Increasing fat at the expense of lactose did not affect MR intake or solid feed intake during the preweaning and weaning periods. However, HF calves tended to consume more solid feed than HL calves during the postweaning period (2.63 ± 0.08 vs. 2.52 ± 0.08 kg/d). Additionally, average daily gain (HF = 0.78 ± 0.02, HL = 0.77 ± 0.02 kg/d) and final BW (HF = 98.8 ± 1.53, HL = 97.7 ± 1.57 kg) were not affected by MR composition. Nevertheless, NEFA concentration was higher in HF calves than in HL calves (0.21 ± 0.01 vs. 0.17 ± 0.01 mmol/L), and glucose concentration was higher in HF calves (6.52 ± 0.23 vs. 5.86 ± 0.23 mmol/L). Under the conditions of this study, HF calves consumed similar amounts of solid feed and grew comparably to the HL calves; however, the isonitrogenous replacement of lactose by fat had evident metabolic effects, such as increased blood NEFA and glucose concentrations.

## Highlights

• Increasing dietary fat in milk replacer did not affect solid feed intake.
• Under experimental conditions, calf performance was not affected.
• Altering macronutrient composition of milk replacer for calves can affect blood metabolites.

## Graphical Abstract

**Figure:**
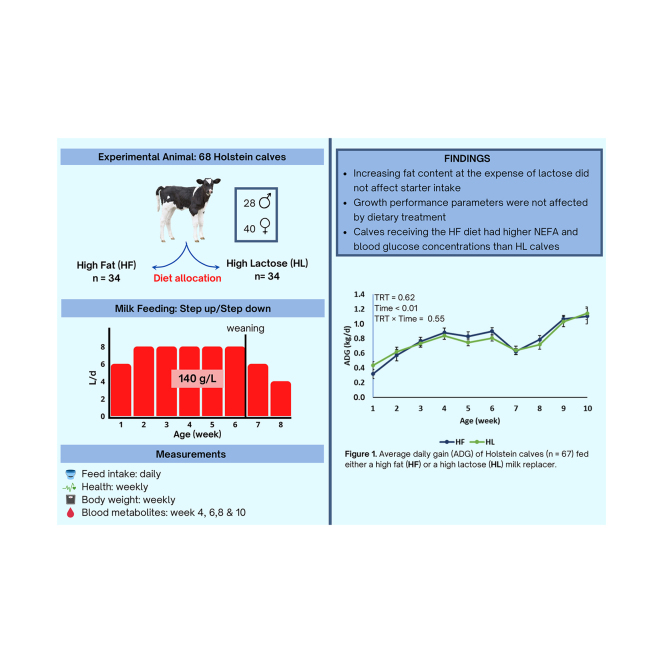
**Summary:** Compared with bovine whole milk, commercial milk replacers commonly contain higher levels of lactose and lower levels of fat. As the industry progresses to higher milk allowances, milk replacer formulations warrant reconsideration. Fat and lactose levels of milk replacers were compared with respect to their effects on feed intake, growth, and blood metabolites in calves fed a high but restricted milk allowance. Increasing fat content in milk replacer did not affect solid feed intake or performance, but blood metabolites were altered by the dietary treatment.

Rearing calves have traditionally been offered ~10% of birth BW by volume of whole milk (**WM**) or milk replacer (**MR**; ~20% CP and ~20% fat DM basis; Jasper and Weary, 2002). With the global trend in the dairy industry moving toward enhanced feeding programs (>20% of birth BW by volume), the feeding of MR higher in protein content (up to 30%, DM basis; Diaz et al., 2001) has been proposed because it can benefit body composition (i.e., Blome et al., 2003). Nevertheless, fat levels remain similar to those in traditional formulations (~20% fat DM; Raeth-Knight et al., 2009). For both situations, lactose levels usually range between 42 and 45% (DM basis; Park, 2009); however, compared with WM (33 to 38% lactose; 30 to 40% fat DM; Pantophlet et al., 2016; Berends et al., 2020), these MR formulations provide a lower dietary energy density, different fat composition in terms of fatty acid profile and triglyceride structure, and a lower energy:protein ratio. Although information is limited, it has been reported that high-fat MR (21.6% of DM; Kuehn et al., 1994), and additional milk or milk solids in the diet (Jaster et al., 1992) can depress solid feed DMI before and after weaning. Studies comparing low-fat MR (<20% fat) with WM (~30% fat), which naturally presents a higher fat content (including a lower lactose content than MR), reported better growth rates (Moallem et al., 2010) and enhanced structural development (Esselburn et al., 2013) when calves were fed WM.

Researchers have been investigating the effects of altering the macronutrient compositions of MR formulations, especially lactose and fat, in an attempt to formulate MR that more closely resembles WM (Amado et al., 2019; Echeverry-Munera et al., 2021; Welboren et al., 2021). Interestingly, more recent studies have reported no differences on BW and ADG of calves fed restricted (Amado et al., 2019) or ad libitum (Berends et al., 2020; Echeverry-Munera et al., 2021) high-fat MR (23% fat DM; vegetable oil) compared with diets containing lower dietary fat levels (17% fat DM; vegetable oil). Notwithstanding, it has been demonstrated that partially replacing lactose with fat (vegetable oil) in MR formulations can increase BW gain as well as gain:ME intake during the first 7 d of life (Welboren et al., 2021). It has also been suggested that greater dietary fat inclusion could be beneficial for glucose homeostasis, because smaller fluctuations in postprandial glucose and insulin concentrations have been observed (Welboren et al., 2021).

Thus, under study conditions and based on previous studies where high-fat diets were offered, we hypothesized that a high-fat diet (1:1 wt/wt lactose by fat exchange) would decrease solid feed consumption; however, calf growth is not expected to be affected by the partial exchange of lactose for fat. The objective of this study was to evaluate the effects of a high-fat MR and a high-lactose MR on growth performance, feed intake, and blood metabolites in rearing calves under isonitrogenous conditions.

This study was conducted at Trouw Nutrition Ruminant Research Facility (Boxmeer, the Netherlands). All experimental procedures and animal care were conducted in accordance with animal welfare legislation and were approved by the animal experimentation committee (DEC Dierexperimentencommissie, Utrecht, approval #2013.III.11.120). A classical power analysis was conducted to determine the number of experimental units needed. The power (1 − β) was chosen to be equal to 80%, and the α-level was 0.05. Solid feed intake was considered the most reliable parameter to determine the power of this study, based on the outcome of a previous study conducted by Echeverry-Munera et al. (2021) with 32 calves allocated to 2 treatments and fed ad libitum milk allowances. At 84 d of age, a standard deviation of 0.130 kg/d was assumed for solid feed intake. The minimal meaningful difference in solid feed intake was 0.190 kg/d. Therefore, the minimal sample size to detect differences at this assumption was calculated to be 34 calves per treatment group when accounting for maximum mortality of 10%.

A total of 68 Holstein-Friesian calves (40 females and 28 males) born at the research facility, with a mean initial BW of 44.1 ± 4.3 kg (mean ± SD) and apparently healthy (no respiratory, heart, skin irregularities, or blindness) were used in this study. Calves were immediately separated from their dams after birth and housed in individual hutches (≥2.5 m^2^) composed of 50% outdoor area and 50% indoor with straw bedding until 70 d of age. Calves received a total of 4 L of pasteurized colostrum (previously frozen and thawed) with a reading of 22% Brix or greater in 2 separate meals. The first colostrum meal (2 L) was given within 1 h of birth, and the second meal (2 L) was given 6 h after birth. Passive immunity was evaluated between 48 and 72 h after birth using the portable Multi-Test Analyzer (DVM Rapid Test II-Multi-Test Analyzer). Calves were blocked based on birth date and parity of the dam (17 blocks, 4 calves of the same sex/block). Within each block, calves were randomly assigned to 1 of 2 treatments: a high-fat (**HF**; 23% fat, 37% lactose, 23.5% CP) or a high-lactose (**HL**; 17% fat, 44% lactose, 23.2% CP) milk replacer. The HF treatment was designed to have similar fat content to WM, and the HL treatment was formulated to resemble commonly available high-lactose MR formulations. Fat and lactose were partially exchanged on a weight per weight (wt/wt) basis, based on spray-dried fat kernels (Trouw Nutrition), with the fat being 35% coconut oil and 65% palm oil. The rest of the MR formulation components remained unchanged and have been previously described by Echeverry-Munera et al. (2021). Because of the weight per weight exchange of lactose for fat, the experimental diets were isonitrogenous but not isoenergetic (HF = 4.7 and HL = 4.4 Mcal/kg of DM), therefore; the CP:ME ratio of the 2 MR was different: 50 versus 53 g of CP/Mcal of ME for HF and HL, respectively.

Calves were fed a fixed allowance of the designated treatment in a teat bucket following a step-up and step-down protocol. Therefore, from d 2 to 7, calves were fed 3 L of MR twice daily; from d 8 to 42, 4 L twice daily; from d 42 to 49, 3 L twice daily; from d 49 to 56, 2 L twice daily; and on d 56 the MR supply was finished. Milk replacer was reconstituted at 140 g of MR/L and offered at 0700 and 1600 h. Calves had constant ad libitum access to water, and fresh calf starter (analyzed composition DM basis: 87.4% DM, 20.4% CP, 24.1% starch, 3.8% fat; ForFarmers B.V.) and dry chopped straw (analyzed composition DM basis: 93.6% DM, 5.4% CP, 1.1% crude fat, 6.2% crude ash, 40.8% fiber; 3- to 7-cm chop length; Ruwvoer Distributiecentrum) were offered daily in separate buckets from d 4 after birth. Calves were weighed with a custom scale (W2000; Welvaarts Weegsystemen) and body measures (wither height, hip height, body barrel, and chest girth) were taken on the day of birth and then once per week. Individual intakes of MR, water, concentrate, and straw were recorded daily by weighing the leftovers.

To evaluate blood metabolites, blood samples were obtained from the jugular vein of a convenience sample of 28 heifer calves (14 calves/treatment). Blood samples were taken in wk 4, 6, 8, and 10 at 1000 h (3 to 4 h after first meal) into one 10-mL EDTA tube for serum, and one 6-mL sodium fluoride (NaF) evacuated tube containing a glycolysis inhibitor for plasma glucose (BD Vacutainer). The EDTA tubes were kept at room temperature for 15 min, and all tubes were centrifuged at 1,500 × *g* for 15 min at 20°C. Samples were stored in 2-mL cryotubes (2 per sample) and stored immediately at −20°C. General calf health was monitored by caretakers daily, and a standard health protocol was followed. All study personnel involved in calf handling and taking measurements were blinded to the treatments.

Blood samples were analyzed at GD Animal Health (Gezondheidsdienst voor Dieren). Serum nonesterified fatty acids (**NEFA**), urea, and cholesterol were analyzed using enzymatic methods. Plasma glucose was determined with an enzymatic method based on hexokinase. Colorimetric methods were used to analyze total bilirubin (dimethyl sulfoxide method), haptoglobin, and albumin (bromocresol green method). Aspartate aminotransferase and gamma-glutamyl transferase were analyzed using enzymatic methods according to the International Federation of Clinical Chemistry and Laboratory Medicine (IFCC) reference procedures for the measurement of catalytic activity concentrations of enzymes at 37°C. Plasma glutamate dehydrogenase was analyzed using an enzymatic method (Deutsche Gesellschaft für Klinische Chemie method).

One bull calf from the HL treatment was excluded from the study due to severe metabolic acidosis and dehydration associated with diarrhea. Data collected from this animal before removal were excluded from the analysis. Continuous variables (i.e., intakes, ADG, feed efficiency, and blood parameters) were analyzed using mixed-model analysis with PROC MIXED procedure of SAS (version 9.4, SAS Institute Inc.). The model included the fixed effects of block, treatment, time, and the interaction between treatment and time. The experimental unit was the calf and time (week) was used as a repeated measure. The autoregressive covariance structure was applied as time points were equally spaced. Initial body weight was used in the model as a covariate for ADG and feed efficiency. Blood parameters and intakes were analyzed without initial BW as a covariate due to a lack of significance of this factor. For BW, the initial and the last measurement (d 70) were used. Body weight data were then analyzed with PROC MIXED with MR treatment as a fixed effect, initial BW as a covariate, and the fixed effect of block. Treatment averages were presented as least squares means (LSM) and standard errors of the mean (SEM). Statistical significance was declared at *P* ≤ 0.05, and trends toward statistical significance were noted when 0.05 < *P* < 0.10.

As a starting point, no differences were observed in plasma IgG (HL = 16.3 ± 1.65 g of IgG/L; HF = 16.0 ± 1.69 g of IgG/L; *P* = 0.62). Initial BW did not differ between the 2 treatments (HF = 44.5 ± 0.78 kg; HL = 43.4 ± 0.80 kg; *P* = 0.33). Final BW was comparable between the 2 treatments, with HF calves being 1.1 kg heavier than HL calves (*P* = 0.62; Table 1). Similarly, ADG was not affected by dietary treatment (*P* > 0.05; Table 1). Feed intake data are presented in Table 1. Milk replacer intake did not differ between the 2 groups during the preweaning (*P* = 0.68) and weaning (*P* = 0.11) periods. Similarly, solid feed intake during the preweaning (*P* = 0.27) and weaning (*P* = 0.84) periods was not affected by dietary treatment. However, during the postweaning period, HF calves tended to consume more starter than HL calves (*P* = 0.08). Additionally, by design, dietary treatments influenced ME intake, with HF consuming more ME than HL calves during the preweaning (*P* = 0.003) and weaning (*P* = 0.05) periods. No differences were detected in straw intake during the study (*P* > 0.05). Nevertheless, feed conversion was comparable between treatments during the whole study (*P* > 0.05). Blood data from 28 heifer calves (14/treatment) are summarized in Table 2. Treatment (*P* = 0.02) and time (*P* = 0.03) effects were observed for NEFA concentration, with greater concentrations in HF than HL calves during wk 6 and 8 (*P* < 0.05; Figure 1A). Similarly, treatment (*P* = 0.04) and time (*P* = 0.002) effects were detected for glucose concentration, with greater concentrations in HF than HL calves during wk 6 and 8 (*P* < 0.05; Figure 1B). No differences (*P* > 0.05) were observed in any of the other blood parameters analyzed.

**Table 1:** Growth performance, intakes, and feed efficiency (LSM ± SEM) for Holstein calves (n = 67) fed milk replacers (MR) differing in dietary energy source during the preweaning (d 1 to 42), weaning (d 43 to 56), and postweaning (d 57 to 70) phases

Item	Treatment^1^				*P*-value^2^		
	HF	SEM	HL	SEM	TRT	T	TRT × T
Growth performance							
Final BW, kg	98.8	1.53	97.7	1.57	0.62	—	—
ADG	0.78	0.02	0.77	0.02	0.62	<0.01	0.55
Intake							
MR, kg of DM/d							
Preweaning	1.01	0.001	1.01	0.01	0.68	<0.01	0.44
Weaning	0.68	0.001	0.67	0.002	0.11	<0.01	0.24
Starter, kg/d							
Preweaning	0.13	0.02	0.12	0.02	0.58	<0.01	0.53
Weaning	0.98	0.05	0.88	0.06	0.19	<0.01	0.32
Postweaning	2.63	0.08	2.52	0.08	0.32	0.01	0.08
ME, Mcal/d							
Preweaning	5.07	0.05	4.85	0.05	0.03	<0.01	0.26
Weaning	5.95	0.16	5.51	0.16	0.05	<0.01	0.61
Postweaning	7.48	0.23	7.15	0.23	0.32	0.02	0.08
Straw, kg/d							
Preweaning	0.02	0.003	0.01	0.003	0.54	<0.01	0.70
Weaning	0.08	0.01	0.08	0.01	0.90	<0.01	0.51
Postweaning	0.11	0.02	0.10	0.02	0.83	0.07	0.10
Mcal/kg gained							
Preweaning	8.11	0.26	7.83	0.26	0.45	<0.01	0.13
Weaning	9.51	0.48	8.66	0.50	0.23	0.55	0.68
Postweaning	7.13	0.32	7.34	0.33	0.65	0.67	0.55

1 Treatments included a high-fat MR (HF; 23% fat, 37% lactose, and 23.5% CP; n = 34) and a high-lactose MR (HL; 17% fat, 44% lactose, and 23.2% CP; n = 33).

2 TRT = treatment effect; T = time effect (week); TRT × T = treatment by time interaction.

**Table 2:** Effect of calf milk replacer (MR) composition on selected blood metabolites (LSM ± SEM) in dairy Heifer calves (n = 28) on wk 4, 6, 8, and 10 at 1000 h

Item	Treatment^1^			*P*-value^2^		
	HF	HL	SEM	TRT	T	TRT × T
Nonesterified fatty acids, mmol/L	0.21	0.17	0.01	0.02	0.03	0.73
Urea, mmol/L	2.90	2.82	0.12	0.65	<0.01	0.22
Glucose, mmol/L	6.52	5.86	0.23	0.04	<0.01	0.37
Cholesterol, mmol/L	2.94	2.87	0.13	0.68	<0.01	0.19
Albumin, g/L	31.4	31.6	0.50	0.71	<0.01	0.92
Haptoglobin, g/L	0.07	0.06	0.01	0.52	0.18	0.74
Total bilirubin, μmol/L	1.80	1.75	0.06	0.52	<0.01	0.46
Glutamate dehydrogenase, IU/L	45.0	51.6	6.73	0.49	<0.01	0.08
Gamma-glutamyl transferase, IU/L	12.6	12.3	1.55	0.91	<0.01	0.63
Aspartate aminotransferase, IU/L	44.7	46.1	1.30	0.46	<0.01	0.38

1 Treatments included a high-fat MR (23% fat, 37% lactose; n = 14; HF) and a high-lactose MR (17% fat, 44% lactose; n = 14; HL).

2 TRT = treatment effect; T = time effect (week); TRT × T = treatment by time interaction.

**Figure 1:**
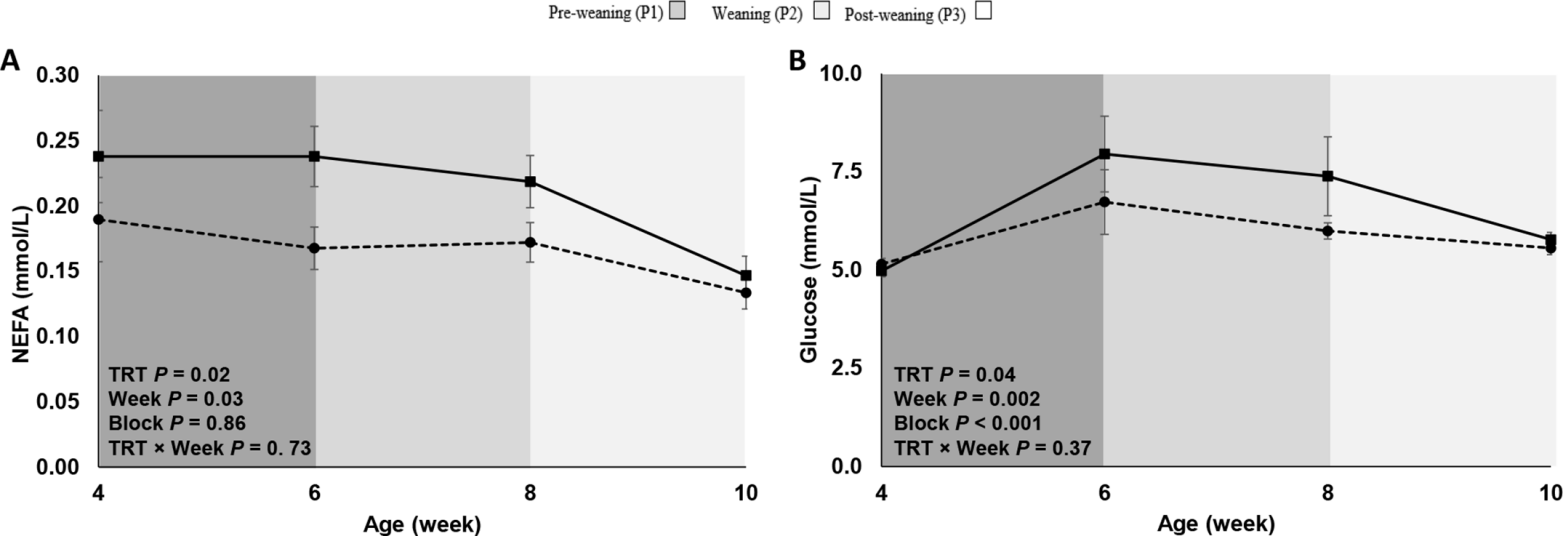
Plasma concentrations of (A) nonesterified fatty acids (NEFA), and (B) glucose measured in heifer calves (n = 28) fed restricted (8 L/d) levels of a high-fat milk replacer (□, HF; 23% fat, 37% lactose, and 23.5% CP; n = 14) or a high-lactose milk replacer (•, HL; 17% fat, 44% lactose, and 23.2% CP; n = 14). Samples were taken on wk 4, 6, 8, and 10 of the study. Treatment (TRT) × time differences at each time point are indicated by *(*P* ≤ 0.05). Standard errors were computed on raw data to better illustrate the observed difference in variability between treatments.

The objective of this study was to evaluate the effects of a high-fat and a high-lactose MR on growth performance, feed intake, and blood metabolites in rearing calves. By design, the dietary treatments in this study provided different amounts of energy due to the partial replacement of lactose by fat (HF = 4.7 and HL = 4.4 Mcal/kg of DM). Despite the macronutrient composition of the MR, calves consumed similar amounts during the preweaning and weaning periods. In a previous study, similar dietary treatments were fed ad libitum, and a 12% (150 g MR/d) reduction in the intake of high-fat MR was observed during the preweaning period (Echeverry-Munera et al., 2021). Therefore, the lack of difference in MR intake in the current study might be attributed to the restrictive nature of the feeding program implemented. Despite the similar intakes, HF calves consumed, on average, more total ME during the preweaning and weaning periods. This difference was a direct consequence of differences in the caloric density of the diets. Although it has been reported that energy intake regulates the consumption of MR in ad libitum-fed calves (Berends et al., 2020), in the current study, this might not be the case due to the restrictive feeding program implemented.

After the initial weeks of life, preweaning starter and liquid feed intake are inversely correlated (Gelsinger et al., 2016). It has been well documented that higher feeding rates of WM or MR might decrease solid feed intake and have adverse effects on rumen development and, consequently, postweaning performance (i.e., Jasper and Weary, 2002). In addition, feeding high-fat diets can have a negative effect on feed intake by exceeding the metabolic capacity of the animal and generating satiety signals (Palmquist, 1994; Choi et al., 1996). Therefore, when implementing enhanced feeding programs, it is important to have an appropriate weaning protocol that allows the animals to maintain the BW gained (Khan et al., 2007). Nevertheless, the lack of difference in starter intake during the preweaning and weaning periods between treatments in the current study was in line with earlier findings (Hill et al., 2008) and suggests that solid feed intake in the presence of enhanced WM or MR is potentially regulated by the rate of rumen development (Meale et al., 2017).

During the postweaning period of the current study, calves in the HF treatment consumed, on average, 110 g/d more starter and greater ME than the HL group, resulting in 1.1 kg greater final BW. These results suggest that calves can grow well under the provision of greater dietary fat levels. Beyond the effects of greater dietary fat inclusion on feed intake and growth, it has been suggested that feeding additional fat usually increases NEFA concentrations in cattle (Choi and Palmquist, 1995). Although the liquid diets in this study were isonitrogenous, they were not isocaloric. Therefore, markers for fat metabolism (e.g., NEFA) were expected to be affected by diet, and thus, were higher during the milk phase (up to wk 8) in heifer calves consuming the high-fat milk replacer. Although Bascom et al. (2007) attributed the elevated NEFA concentrations in calves fed a high-fat MR (33% vs. 16% fat) to the fat source in the diet (edible lard as opposed to milk fat), in the current study, the increased NEFA concentrations in HF calves seemed to be a direct result of the greater inclusion of fat in the diet. These results agree with more recent studies where similar compositions were fed ad libitum (Berends et al., 2020; Echeverry-Munera et al., 2021).

Plasma glucose concentration in heifer calves was also affected by dietary treatment. During the preweaning period, glucose is obtained primarily from the consumption of MR and digestion of lactose. However, during the weaning and postweaning periods, the liver nutrient supply starts to change from glucose to short-chain fatty acids from ruminal fermentation in the ruminating calf (Suarez-Mena et al., 2017); therefore, plasma glucose concentration is expected to decrease due to shifts in the hepatic metabolic activity of the calf from glycolytic to gluconeogenic. Although HL calves were expected to have greater glucose concentrations due to the nature of the diet, calves consuming the HF treatment showed higher blood glucose levels. Nevertheless, in the current study, blood samples were taken from the heifer calves after the first month of life (at wk 4), which differs from other studies (Welboren et al., 2021) that have looked at the effect of MR composition earlier in life. Although sampling was performed 3 to 4 h after MR feeding on average, calves in the current study still did not experience hyperglycemia (blood glucose concentrations >8.3 mmol/L; Hostettler-Allen et al., 1994). While differences were detected for some blood metabolites (i.e., NEFA and glucose), there is the possibility of a type I error because metabolic indicators were measured using a convenience sample size, rather than a calculated formal sample size.

Increasing fat content at the expense of lactose resulted in an increase of 6% in energy density of the HF treatment; as a consequence, ME intake was different throughout the study. Regardless, under the experimental conditions, dietary composition did not affect MR acceptance or starter intake. Growth rate and BW were similar despite energy intake differences, but we did not evaluate body composition. Balancing the lactose-to-fat ratio of MR toward that of WM by increasing fat and reducing lactose could have metabolic effects, as shown by greater NEFA and blood glucose concentrations in HF calves.
